# Supplementary material for: Harnessing virus flexibility to selectively capture and profile rare circulating target cells for precise cancer subtyping
Source: Nat Commun. 2024 Jul 12;15:5849. doi: 10.1038/s41467-024-50064-y (PMC11239949; doi:10.1038/s41467-024-50064-y)
Supplement: Supplementary file 1 — Supplementary Information [file 41467_2024_50064_MOESM1_ESM.docx]

**Supporting information**

**Harnessing virus flexibility to selectively capture and profile rare circulating target cells for precise cancer subtyping**

*Hui-Da Li ^a^, Yuan-Qiang Chen ^b^, Yan Li ^c^, Xing Wei ^a^, Si-Yi Wang ^a^, Ying Cao ^a^, Rui Wang ^a^, Cong Wang ^d^, Jing-Yue Li ^d^, Jian-Yi Li ^d^*, Hong-Ming Ding ^b^*, Ting Yang ^a^*, Jian-Hua Wang ^a^, Chuanbin Mao ^e^**

^a^ Research Center for Analytical Sciences, Department of Chemistry, College of

Sciences, Northeastern University, Box 332, Shenyang 110819, China.

^b^ Center for Soft Condensed Matter Physics and Interdisciplinary Research, Soochow University, Suzhou 215006, China.

^c^ Department of Periodontology, The Second Affiliated Hospital, College of Medicine, Zhejiang University, Hangzhou, 310009, PR China

^d^ Department of Breast Surgery, Liaoning Cancer Hospital & Institute, Cancer Hospital of China Medical University, Shenyang 110042, China.

^e^ Department of Biomedical Engineering, The Chinese University of Hong Kong, Hong Kong SAR, China.

***Corresponding author.**

*E-mail address:

yangting@mail.neu.edu.cn (T. Yang)

sjbreast@yeah.net (J. Li)

dinghm@suda.edu.cn (H.-M. Ding)

cmao@cuhk.edu.hk (C. B. Mao)

**1. Supplementary experimental information**

**Supplementary Table 1.** The sequences of aptamers and primers.

**Apparatus**

**2. Supplementary results**

**S1.** Azide modification of 6His-M13 phage: modification efficiency calculation and reaction condition optimization.

**S2.** Aptamer loading amount on M13 with different stiffness.

**S3.** Comparisons between flexible M13, rigid M13 and aptamers: cell binding affinity and topological morphology.

**S4.** M13 anchoring on the Ni-IDA grafted glass slides.

**S5.** Aptamer decoration on the epoxy glass slides.

**S6.** Construction of the coarse-grained (CG) models in DPD simulations.

**S7.** Aptamer S2.2 targeting ability towards MUC1^+^ cells.

**S8.** Experimental optimization for CTC capture.

**S9.** Experimental optimization for CTC release and comparisons of the release performance between A-f-M13-MB and A-MB strategy in buffer/blood.

**S10.** Evaluation of the phenotype drifting and cell migration ability of CTCs before and after isolation by A-f-M13-MBs and re-culture.

**S11.** Immunofluorescence staining of different breast cell lines.

**S12.** Clinical sample information.

**S13.** Isolation of CTC mixtures with different EMT subphenotypes by M13-MBs bearing Y-shaped DNA scaffold.

1. **Supplementary experimental information**

**Supplementary Table 1: The sequences of aptamers and primers.**

| Name | Sequence（5'- 3'） |
| --- | --- |
| DBCO-Apt | DBCO-TTTTTGCAGTTGATCCTTTGGATACCCTGG |
| FAM-Apt-DBCO | DBCO-TTTTTGCAGTTGATCCTTTGGATACCCTGG-FAM |
| NH_2_-Apt | NH_2_-TTTTTGCAGTTGATCCTTTGGATACCCTGG |
| FAM-Apt-NH_2_ | NH_2_-TTTTTGCAGTTGATCCTTTGGATACCCTGG-FAM |
| 6H-Tag1 | GTACCTTTCTATTCTCACTCTCATCATCATCATCATCATTCCTCCAAACTGCAGTC |
| 6H-Tag2 | GGCCGACTGCAGTTTGGAGGAATGATGATGATGATGATGAGAGTGAGAATAGAAAG |
| Y_1_ | CACGCATAGCCTTTGCTCCTCGTCTGGAACGTCGCAGCTTTAGTTCTGGGCCTATGCGTGTTTTTTGTAGTCGGTACCTAAGACTTCTGAGCATGCACTGAC |
| Y_2_ | CACTACAGAGGTTGCGTCTGTCCCACGTTGTCAT  GGGGGGTTGGCCTGTTTTTTGTCAGTGCATG  CTCAGAAGAACTCACGTGACG |
| Y_3_ | DBCO-ATTGCGTATGTCACGTCACGTGAGTTCGTCTTAGGT  ACCGACTAC |

**Apparatus**

The modification efficiency of N_3_-M13 phage was analyzed by Q-TOF LC/MS (Agilent Technologies, 1260-6540) with a 0.1 mL min^-1^ liquid speed and the mass spectra were acquired with a range of 5000−6000 m/z. The fluorescence (FL) measurements for binding constants were carried on a microplate reader (Synergy H1, BioTek, USA). The cell topological structure was observed by a Hitachi SU8010 scanning electron microscopy (SEM) at 200 kV (Hitachi, Japan). The flexible M13 anchored on microbeads were observed using transmission electron microscope (JEM-2100 Plus, JEOL, Japan). The cell enumeration and the fluorescence observation of live-dead cells were conducted using a forward microscope (Olympus, BX53M). A Bruker Dimension icon atomic force microscopy (AFM, Bruker, Germany) was used for the imaging of M13 phages and the measurement of their Young's modulus and stiffness. The fluorescence images of aptamer or antibody labelled cells were observed using confocal laser scanning microscope (CLSM, FV 1200, Olympus, Japan). The cell migration ability before and after isolation and re-culture was evaluated by Live-cell Station (CytoSMART Lux3, Axion BioSystems, USA).

**2. Supplementary results**

**S1. Azide modification of 6His-M13 phage: modification efficiency calculation and reaction condition optimization.**

The modification efficiency of azide was calculated according to the external standard method. Briefly, we first measured the MS response of pVIII protein (m/z 5238) at different amounts of 6His-M13 to obtain the calibration curve of the MS response (*Y*) against the concentration of 6His-M13 (*X*) as: *Y*=14.865*X*+0.136. Therefore, by bringing its concentration into the calibration curve, its theoretical MS response was calculated to be 181230. Thereafter, the MS response of 1.21 x 10^9^ pfu of N_3_-M13 (amount obtained by titering) was measured to be 25542 (Supplementary Fig. 1). As the decrement in MS response at m/z 5238 was due to the modification of N_3_, the modification efficiency of N_3_ can be estimated as follows:

$$\frac{\text{1}\text{81230-25542}}{\text{1}\text{81230}}\text{×1}\text{00\%=85.}\text{91}\text{\%}$$

By using the same method described above, we obtained the modification efficiency of N_3_ at different reaction ratios between N_3_-PEG-NHS and the phages, and investigated the optimal reaction ratio. The stability of the resultant N_3_-M13 was also investigated in terms of the modification efficiency of N_3_. As shown in Supplementary Fig. 2, the optimal ratio between N_3_-PEG-NHS and NH_2_-M13 was set as 500: 1, and the resultant N_3_-M13 was found to be stable for at least one week.


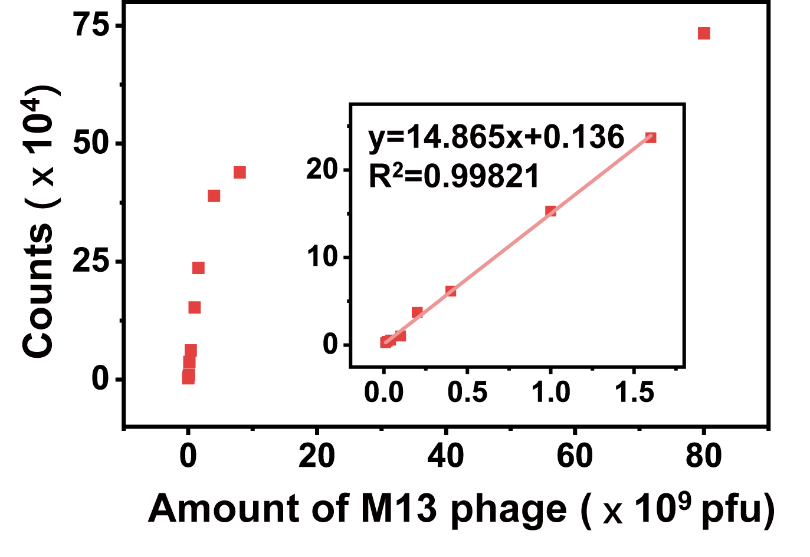


**Supplementary Fig. 1.** LC-MS response (m/z 5238) of different amount of 6His-M13 phage. Source data are provided as a Source Data file.


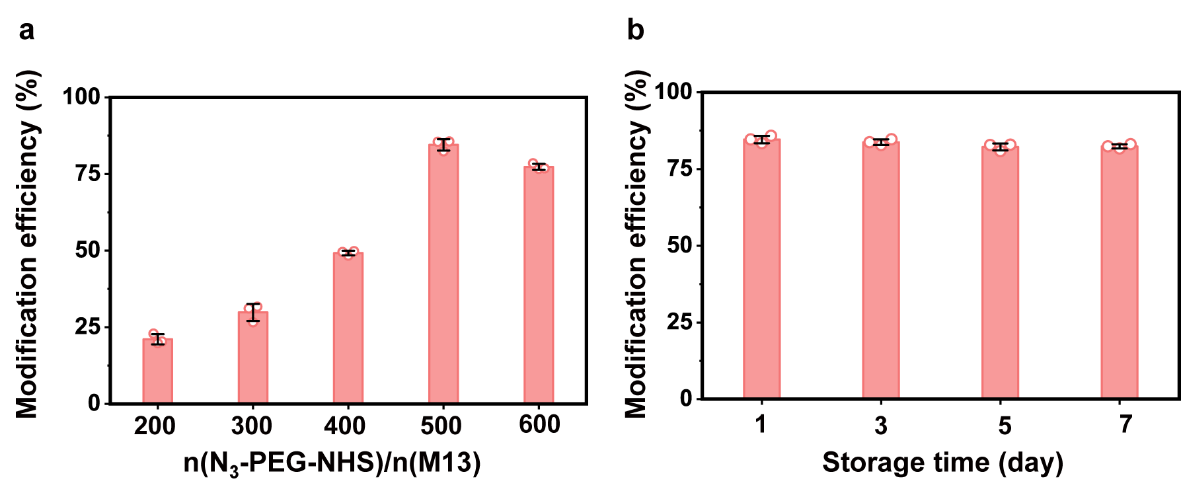


**Supplementary Fig. 2.** **a**, Optimization of the reaction ratio between N_3_-PEG-NHS and M13 (*n* = 3 independent experiments). **b**, Stability of N_3_-M13 within 7 days (*n* = 3 independent experiments). Error bars represent the mean ± s.d. Source data are provided as a Source Data file.

**S2. Aptamer loading amount on M13 with different stiffnesses.**

To assess the amount of the loaded aptamers on N_3_-M13 phages with different stiffnesses, N_3_-M13 was first anchored on MBs and then reacted with FAM-labeled DBCO-aptamer (FAM-Apt-DBCO) for fluorescence measurement^1^. The fluorescence intensity of the supernatant after reaction was measured to obtain the amount of the unreacted FAM-Apt-DBCO (Supplementary Fig. 3). Therefore, the amount of the loaded aptamers on N_3_-M13 phages could be calculated by subtraction.

Taking the calculation of the aptamer loading on untreated M13 phage as an example, 5 x 10^10^ pfu (determined by tittering) of untreated N_3_-M13 was first anchored on MBs (2.5 mg mL^-1^, 1 mL). After the anchoring step, we collected the supernatant to get the number of M13 phages that were not anchored on MBs by titer. The number of M13 phages anchored on MBs can therefore be obtained by subtraction. Meanwhile, we estimated the amount of MBs in 100 μL of suspension to be 2.567 x 10^5^ by microscope observation. Therefore, we can know how many M13 phages were anchored on each MB (~16714 phages for each MB). Afterwards, the N_3_-M13-MBs were further reacted with excessive FAM-labeled DBCO-aptamer (FAM-Apt-DBCO). We measured the fluorescence intensity of the supernatant after reaction to obtain the amount of the unreacted FAM-Apt-DBCO, so the amount of the loaded aptamers (1.04 x 10^7^) on N_3_-M13-MBs can be obtained by subtraction. In this way, the amount of aptamers on each untreated M13 phage can be readily obtained (1.04 x 10^7^ /16714=622). Similarly, the amount of loaded aptamers on PFA-M13 and EtOH-M13 can also be calculated, and the results are shown in Supplementary Table 2.

As shown in Supplementary Table 2, the amount of loaded aptamers on flexible M13 and PFA-M13 was almost identical, whereas that on EtOH-M13 was slightly lower. Therefore, PFA-M13 was further chosen as the model for rigid M13 for fair comparison. Since in the case of PFA-M13, phages were hardened first and then loaded with aptamers, the aptamers were not denatured by PFA.


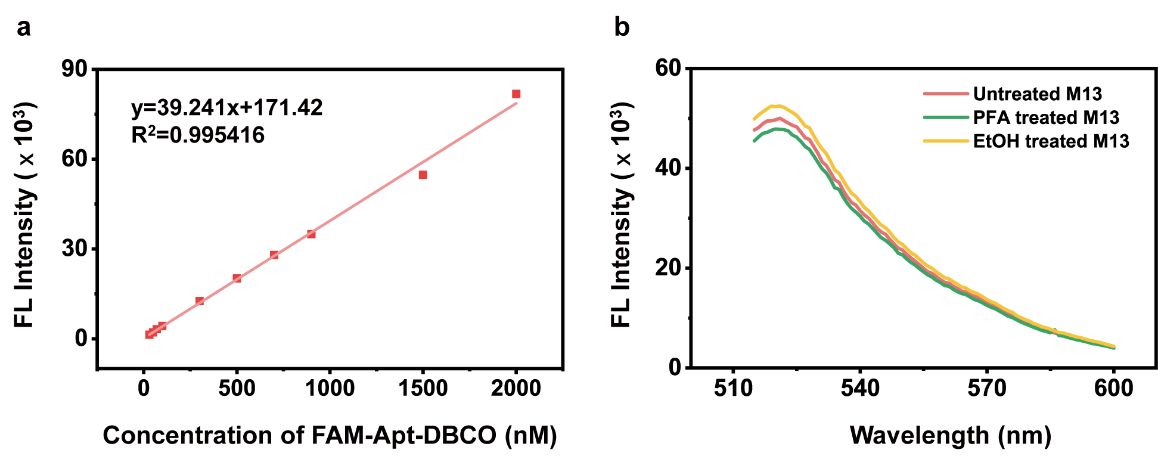


**Supplementary Fig. 3.** **a**, The calibration curve of FAM-aptamer. **b**, The fluorescence spectra of FAM-aptamer loaded M13 with various stiffness. Source data are provided as a Source Data file.


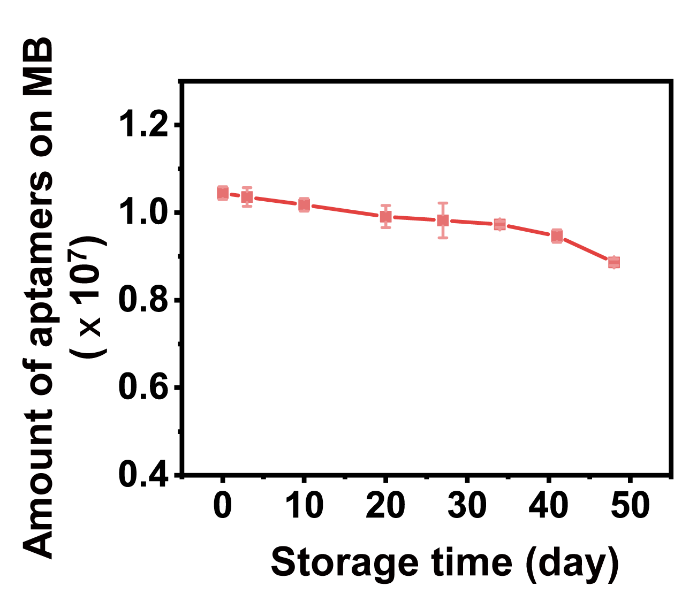


**Supplementary Fig. 4.** The amount of aptamer remaining on each A-f-M13-MB after storage for 0-48 days (*n* = 3 independent experiments). Error bars represent the mean ± s.d. Source data are provided as a Source Data file.

**Supplementary Table 2: The number of loaded aptamers on each MB or M13 for flexible M13 and rigid M13.**

|  | Untreated M13 | PFA-treated (rigid) M13 | EtOH-treated (rigid) M13 |
| --- | --- | --- | --- |
| Amount of aptamers on each MB ( x 10^7^) | 1.04 | 1.26 | 0.7 |
| Number of aptamers on each M13 | 622 | 756 | 444 |

**S3. Comparisons between flexible M13, rigid M13 and aptamers: cell binding affinity and topological morphology.**

In order to compare the CTC binding affinity (MCF-7 cell), either aptamer loaded M13 or free aptamer were immobilized on MBs for conducting the binding affinity assay. As shown in Fig. 4a, the MCF-7 binding constant *K*_d_ by A-f-M13-MB, A-r-M13-MB and A-MB was calculated to be 1.09±0.14 pM, 24.80±2.33 pM and 20.96±2.03 nM, respectively. As the *K*_d_ by free aptamer was 260.09±31.22 nM, the increased cell binding affinity of A-MB was mainly attributed to the multivalent effect. As for M13 nanofibers, the flexible M13 showed a 22-fold increment in binding affinity compared to rigid M13, indicating the flexibility could help M13 nanofibers to adaptively fit the distribution of target receptors on the cell surfaces, significantly enhancing the CTC binding affinity.

For the assessment of the topological effect on the cell capture, SEM images of the captured MCF-7 cells on A-f-M13-slide, A-r-M13-slide and A-slide were compared. As indicated in Supplementary Fig. 5a-c, the smooth surface of apt-slide barely induced pseudopodia structures of MCF-7. On the contrary, both A-f-M13-slide and A-r-M13-slide induced plenty of pseudopodia structures, which was mainly due to the nanostructure of M13 on the slide surface. The induced pseudopodia increased the contact area between the cells and the slide surface, thus contributing to the enhanced cell capture. It was also clear that the topological effect was almost the same for A-f-M13-slide and A-r-M13-slide, and thus the enhanced CTC binding by the former mainly arose from the flexibility of M13 in it.


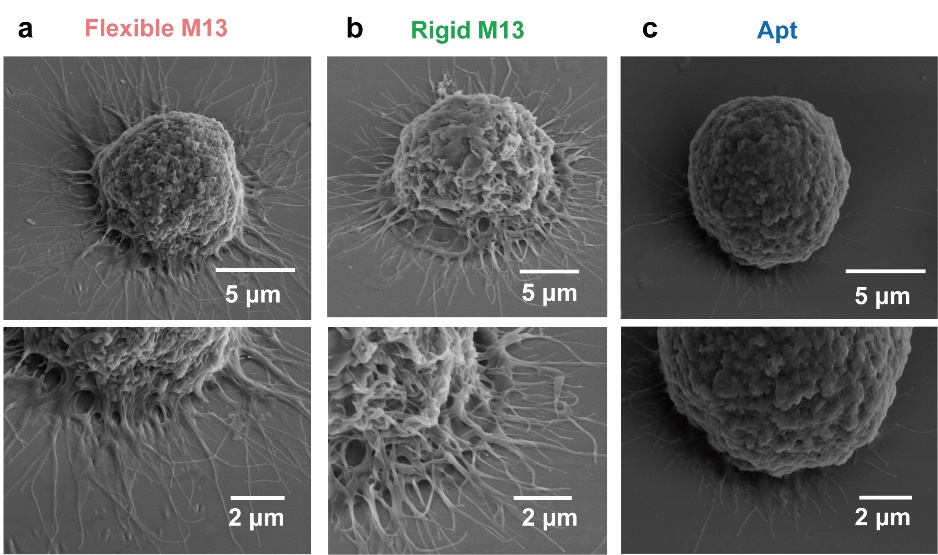


**Supplementary Fig. 5.** The topological effect SEM images of (**a**) A-f-M13-slide, (**b**) A-r-M13-slide and (**c**) A-slide for MCF-7 cells. The representative images are shown from three independent repeats.

**S4. M13 anchoring on the Ni-IDA grafted glass slides.**

For verifying the successful anchoring of M13 on Ni-IDA grafted glass slides, the 6His-M13 was pre-stained with rhodamine dyes in a carbonate solution for 2 h and purified by PEG/NaCl precipitation^2, 3^. Subsequently, the rhodamine-stained 6His-M13 phages were reacted with Ni-IDA modified glass slides for 2 h, and the glass slide was then repeatedly washed for observation under a fluorescence microscope (Olympus, BX53M). The wild type M13 phage and the bare slide were used as control. As shown in Supplementary Fig. 6a-c, 6His-M13 was successfully anchored on the surface of Ni-IDA grafted glass slides through the chelation between 6His and Ni-IDA. We then anchored N_3_-M13 on the Ni-IDA grafted glass slides followed by the loading of FAM-Apt-DBCO. The aptamers were readily conjugated on the N_3_-M13 phages on the glass slides (Supplementary Fig. 6d-f).


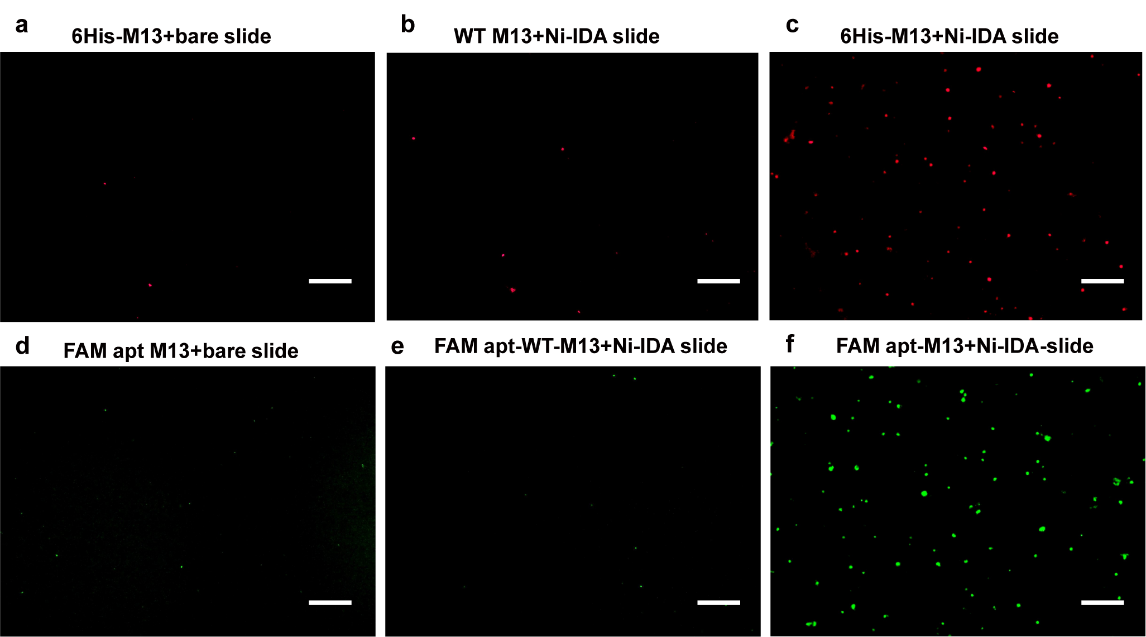


**Supplementary Fig. 6. a-c**, The fluorescent images of rhodamine-stained 6His-M13 anchored on Ni-IDA grafted slide and controls. (**d-f**) The fluorescent images of FAM-aptamer modified M13-slide and controls. Scale bar: 20 μm. The representative images are shown from three independent repeats.

**S5. Aptamer decoration on the epoxy glass slides.**

The successful decoration of aptamers on the epoxy glass slides were confirmed with the fluorescent image of the FAM-aptamer loaded glass slide shown in Supplementary Fig. 7.

**
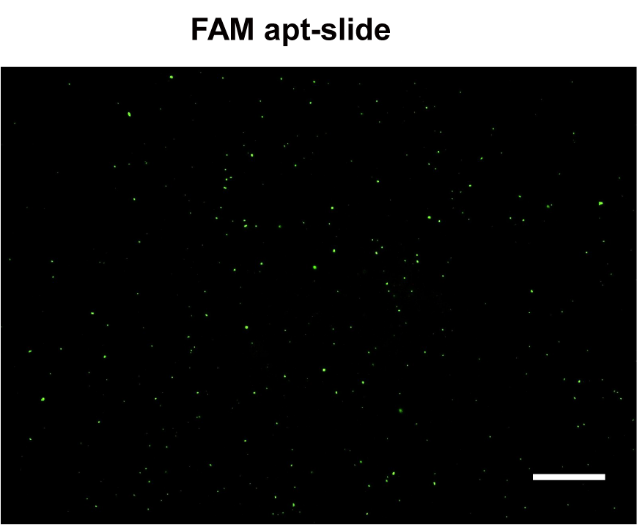
**

**Supplementary Fig. 7.** The fluorescent image of FAM-aptamer loaded glass slide. Scale bar: 20 μm. The representative images are shown from three independent repeats.

**S6. Construction of the coarse-grained (CG) models in DPD simulations.**

**
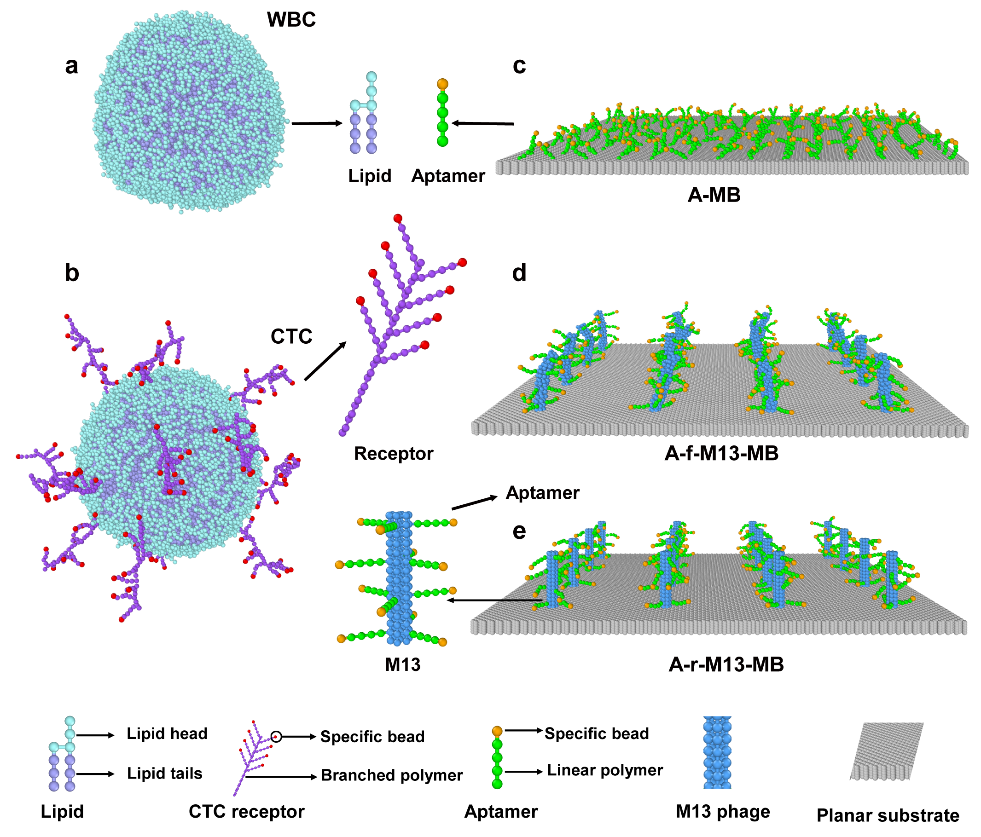
**

**Supplementary Fig. 8.** Illustration of the CG model in DPD simulations. (**a**) The WBC cell is modeled as a lipid vesicle, where the lipid has two tails (iceblue beads) and one head (cyan beads). (**b**) The CTC cell is modeled as a lipid vesicle with some receptors on its surface, where the lipid is the same as that in the WBC and the receptor is modeled as the branched polymer (purple beads) with one specific bead (red bead) at the terminal of each branch. (**c**) The A-MB is modeled as a planar substrate (gray bead), and the aptamer was modeled as the linear polymer (green bead) with one specific bead (orange) at the solvent exposed end. (**d**) The A-f-M13-MB is modeled as a planar substrate with sixteen cylinders (with each cylinder modeling M13, blue bead) coated on its surface. The neighboring beads in the cylinder is connected by a harmonic bond to ensure its integrality. (**e**) The A-r-M13-MB is the same as the A-f-M13-MB except that the cylinder is treated as a rigid body in the simulations.

**S7. Aptamer S2.2 targeting ability towards MUC1^+^ cells.**

Different cell lines were stained with FAM labeled aptamer S2.2 to confirm its targeting ability towards MCF-7. As shown in Supplementary Fig. 9, MUC1^+^ cell lines including MCF-7 and A549 were readily stained by aptamer S2.2 while MUC1^-^ cell lines (HepG2 and SK-Hep-1 cells) cannot, indicating good selectivity of aptamer S2.2 for MUC1.


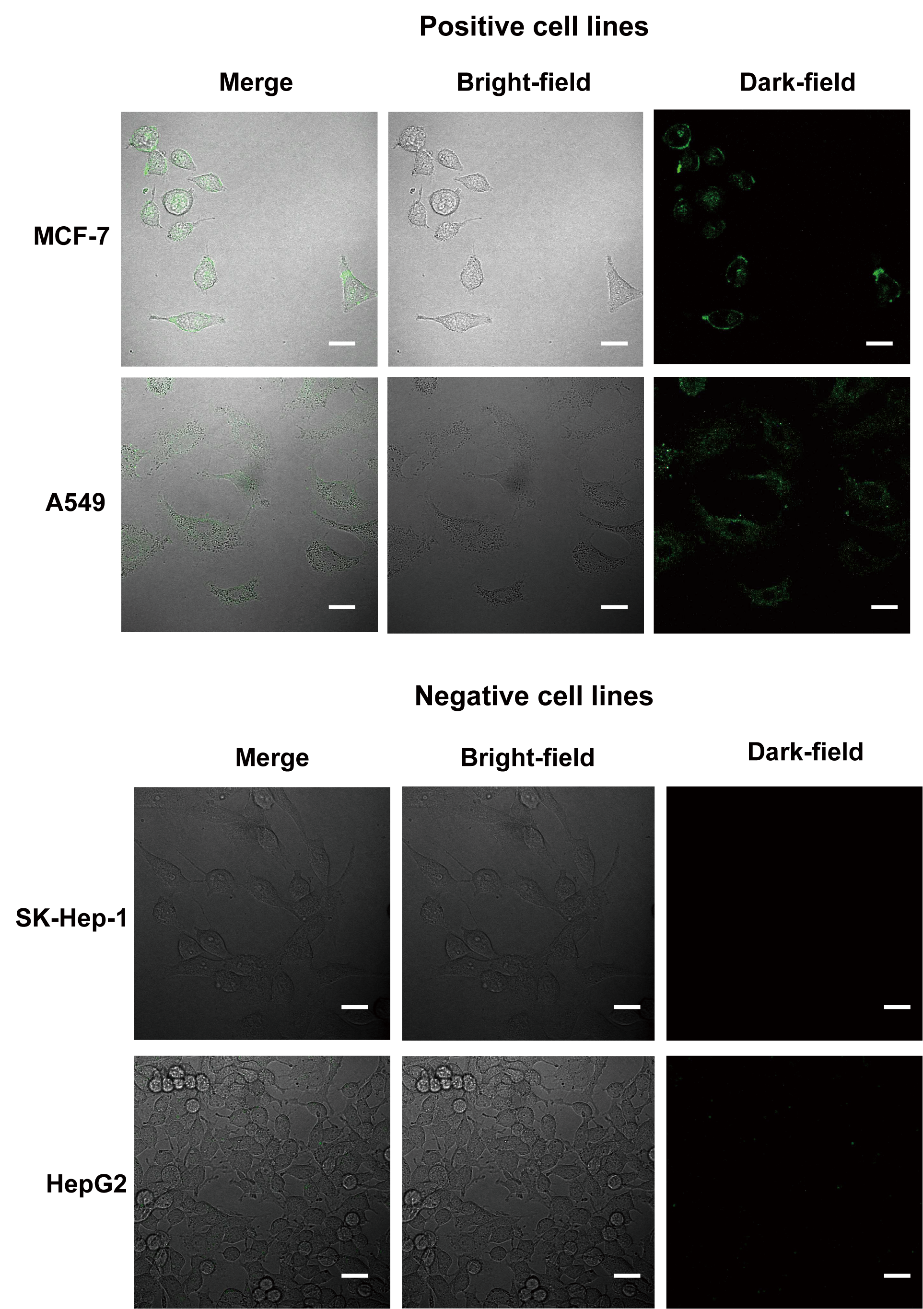


**Supplementary Fig. 9.** The fluorescence images of different cancer cells (MCF-7, A549, SK-Hep-1, HepG2) observed under CLSM. Scale bar: 20 μm. The representative images are shown from three independent repeats.

**S8. Experimental optimization for CTC capture.**

To optimize the CTC capture performance, various parameters, including aptamer loading order, click reaction time, aptamer concentration, N_3_-M13 concentration and incubation time for CTC capture were investigated. Optimal experimental conditions could be concluded from Supplementary Fig. 10 and 11 as follows: To prepare A-f-M13-MB, 6His-M13 phage was firstly anchored on MBs and then reacted with aptamer via click reaction for 6 h; the final reacting aptamer concentration was 1500 nM, and M13 phage input amount was 10^10^ pfu, and the MBs were incubated with cells for 30 min for CTC capture.


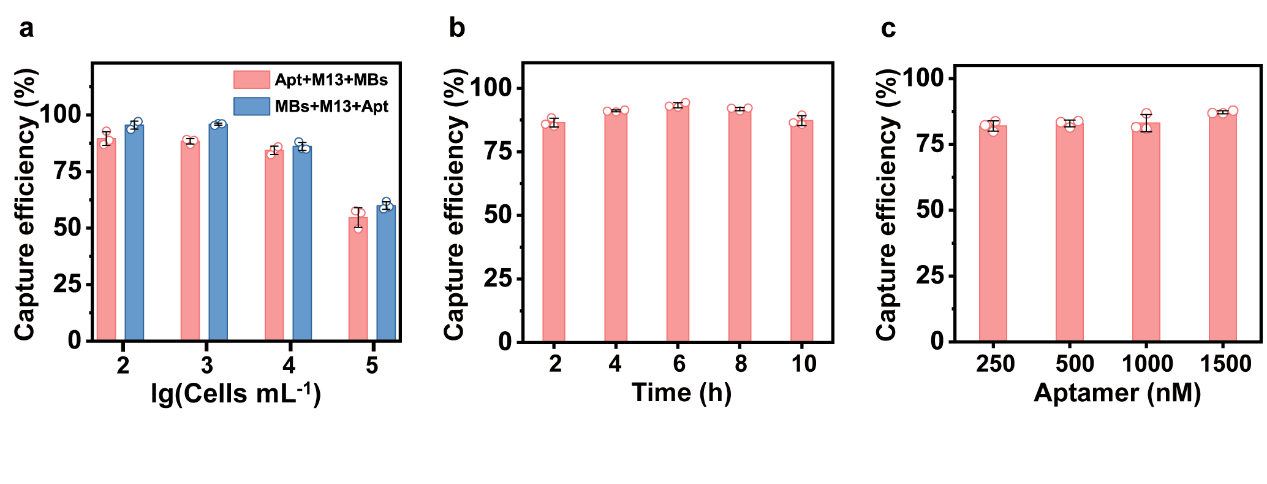


**Supplementary Fig. 10.** Optimization of different strategies (In the Apt+M13+MBs strategy, M13 phage was first reacted with aptamer then anchored on MBs. In MBs+M13+Apt strategy, M13 phage was firstly anchored on MBs and then reacted with aptamer) for the preparation of A-f-M13-MB (**a**), click reaction times (**b**) and aptamer concentrations (**c**). *n* = 3 independent experiments in all cases. Error bars represent the mean ± s.d. Source data are provided as a Source Data file.


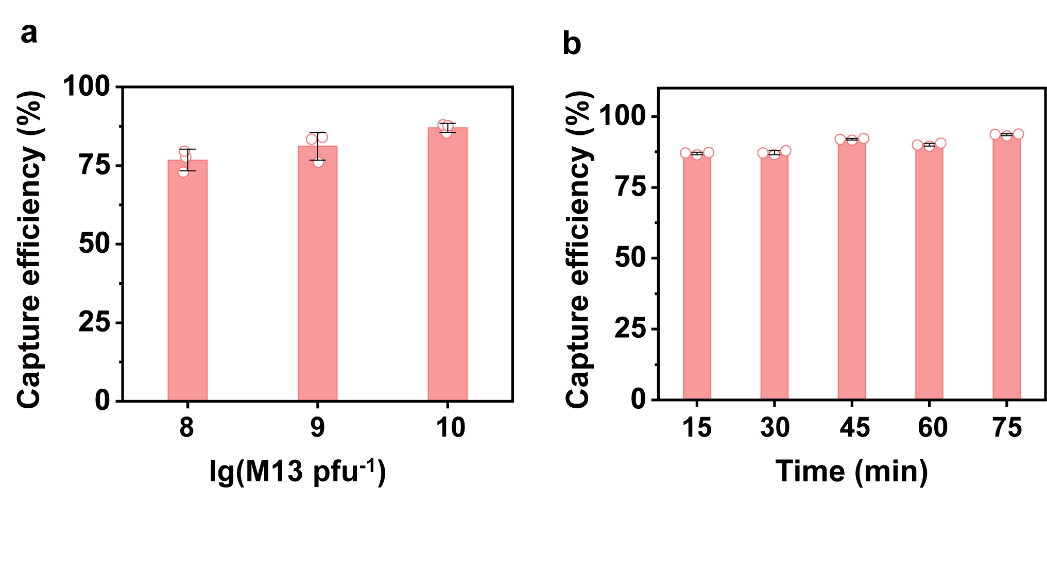


**Supplementary Fig. 11.** Optimization of (**a**) M13 phage input amount and (**b**) incubation times for CTC capture. *n* = 3 independent experiments in all cases. Error bars represent the mean ± s.d. Source data are provided as a Source Data file.

**S9. Experimental optimization for CTC release and comparisons of the release performance between A-f-M13-MB and A-MB strategy in buffer/blood.**

The release of CTC relied on the digestion of aptamers by DNase I, which needs a sufficient enzyme concentration and digestion time. As shown in Supplementary Fig. 12, a DNase I concentration of 100 U mL^-1^ and a release time of 20 min were good enough to get satisfied release efficiency and thus were chosen as the optimum release condition.


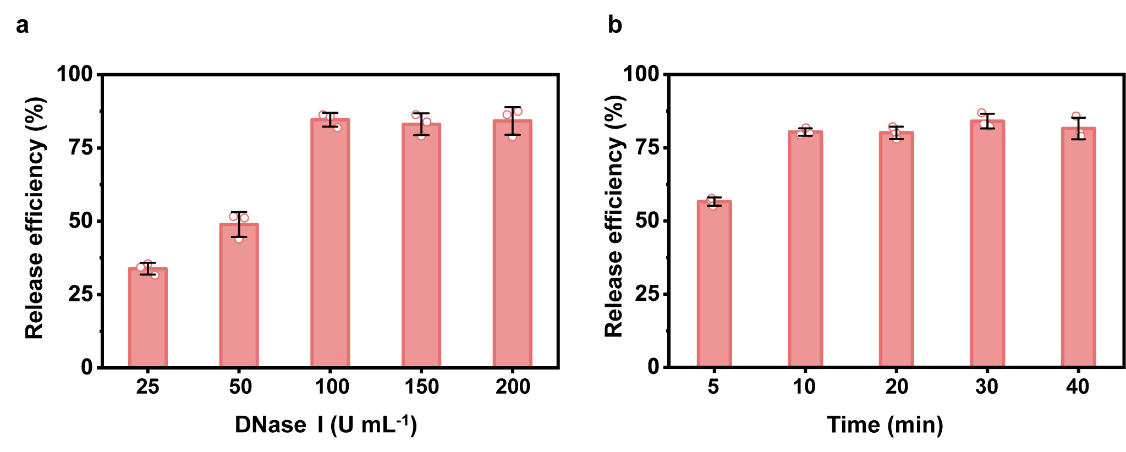


**Supplementary Fig. 12.** Optimization of concentration of DNase I (**a**) and incubation time (**b**). *n* = 3 independent experiments in all cases. Error bars represent the mean ± s.d. Source data are provided as a Source Data file.


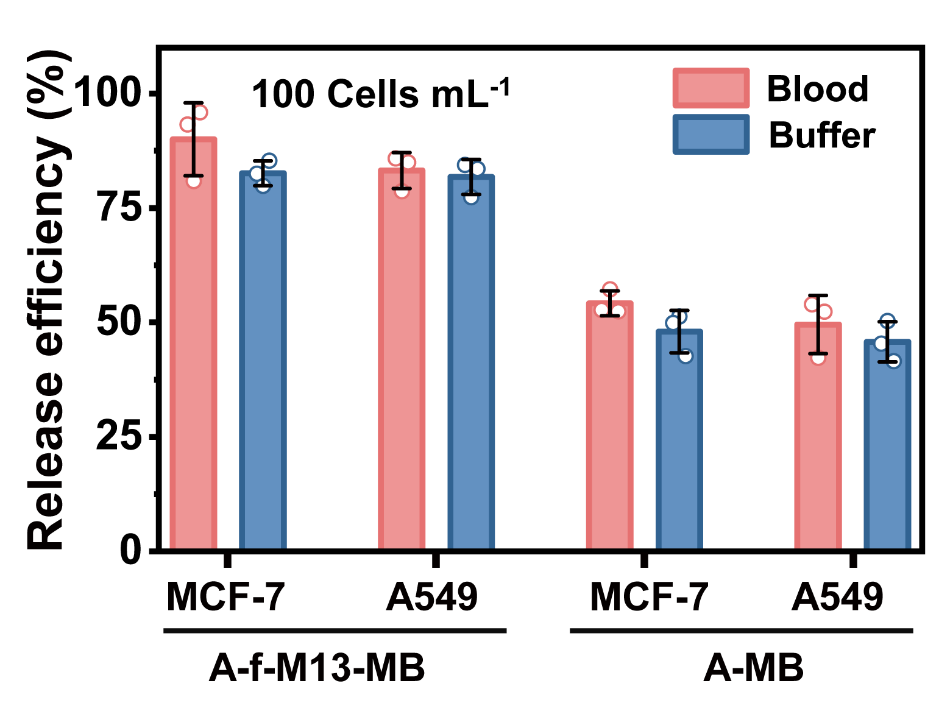


**Supplementary Fig. 13.** The release performance of A-f-M13-MB and A-MB in buffer and blood (*n* = 3 samples). Error bars represent the mean ± s.d. Source data are provided as a Source Data file.

**S10. Evaluation of the phenotype drifting and cell migration ability of CTCs before and after isolation by A-f-M13-MBs and re-culture.**

In order to guarantee the released CTC to be used for downstream applications, such as in vitro CTCs culture or mouse transplantation xenograft construction, the released CTCs should have no obvious phenotype drifting and identical cell activity compared to original CTCs. In order to verify this, MCF-7 cells were first isolated by A-f-M13-MBs and re-cultured for 12 days, and the expression level of estrogen receptor protein (ER, feature biomarker of MCF-7 cell) were evaluated by immunofluorescent imaging and WB analysis. The cell migration ability was evaluated in terms of mean migration distance and mean migration speed. The results are shown in supplementary Fig. 14.


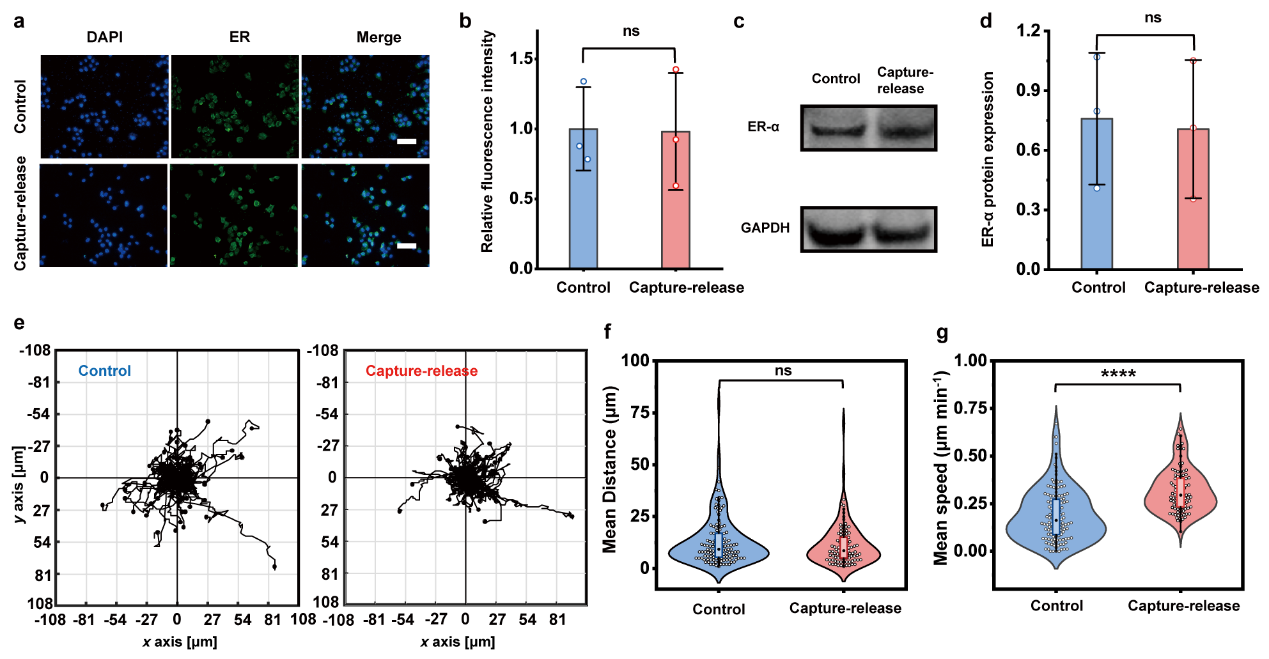


Supplementary Fig. 14 The phenotype drifting profile and comparison in migration ability of CTCs before and after isolation by A-f-M13-MBs and re-culture. The fluorescence images (a) and relative fluorescence intensity (b) of MCF-7 cells before (labeled as “control”) and after isolation by A-f-M13-MBs and being re-cultured for 12 days (labeled as “Capture-release”). Cells were stained by DAPI and anti-ER antibody. *n* = 3 samples for both the control and capture-release groups. Error bars represent the mean ± s.d. p=0.951. ns indicates non-signiﬁcance (p>0.05). The two groups showed no significance, indicating identical ER expression level. Scale bar: 50 μm. (c) Western blotting analysis of ER in MCF-7 cells before (labeled as “control”) and after isolation by A-f-M13-MBs and being re-cultured for 12 days (labeled as “Capture-release”). GAPDH was used as an internal reference. (d) Quantitative analysis of the expression level of ER based on relative gray level of ER band with respect to that of GAPDH in western blotting results. *n* = 3 samples for both the control and capture-release groups. Error bars represent the mean ± s.d. p=0.860. ns indicates non-signiﬁcance (p>0.05). The two groups showed no significance, indicating identical ER expression level. (e) The migration pathway images of MCF-7 cells before (labeled as “control”) and after isolation by A-f-M13-MBs and being re-cultured for 12 days (labeled as “Capture-release”). *n* = 138 samples for the control group, and n=100 samples for the capture-release group. Error bars represent the mean ± s.d. Quantitative analysis of the mean distance (f) and mean speed (g) of cells. For (f), *n* = 134 samples for the control group, and n=98 samples for the capture-release group. Error bars represent the mean ± s.d. p=0.222. ns indicates non-signiﬁcance (p>0.05). For (g), *n* = 138 samples for the control group, and n=100 samples for the capture-release group. Error bars represent the mean ± s.d. p= 1.0048 x 10^-12^. **** p<0.0001. The mean distance of the two groups of cells showed no significance, but the migration speed of the cells after isolation and re-culture was a little faster than the control cell, indicating higher viability. For (g) and (f), unpaired two-sided Student’s t-test were used. The central dot is the median; box bounds are 25^th^ and 75^th^ percentiles, upper and lower limits of whiskers are 1.5× interquartile ranges. Values outside of the upper and lower limits are deﬁned as outliers. The representative images in (a) are shown from three independent repeats. The full uncropped scans of the WB images in (c) are provided in the Source Data file. Source data are provided as a Source Data file.

**S11. Immunofluorescence staining of different breast cell lines.**

The difference in the protein expression among four types of breast cell lines was investigated by subjecting the cells to antibody immunofluorescence staining, and the results were shown in Supplementary Fig. 15-18. The distinct protein expression feature made it feasible to use these cells as model cells to determine the various subtypes of breast cancers, i.e., MCF-7: luminal model cell, MUC1^+^/HER2^-or+^/ER^+^; SK-BR-3: HER2 model cell, MUC1^+^/HER2^+^/ER^-^; MDA-MB-231: triple-negative model cell, MUC1^+^/HER2^-^/ER^-^; MCF-10A: normal mammary epithelial model cell, MUC1^-^/ HER2^-^/ER^-^ ( Alexa Fluor® 488 Anti-MUC1 antibody (Cat. #: ab196443, clone: EPR1023, 1:50 dilution, Abcam, USA, <https://www.abcam.cn/products/primary-antibodies/alexa-fluor-488-muc1-antibody-epr1023-ab196443.html>), Alexa Fluor® 488 Anti-HER2 antibody (Cat. #: ab237060, clone: EP2324Y, 1:100 dilution, Abcam, USA, <https://www.abcam.cn/products/primary-antibodies/alexa-fluor-488-erbb2--her2-phospho-y877-antibody-ep2324y-ab237060.html>), Alexa Fluor® 647 Anti-Estrogen Receptor alpha antibody (Cat. #: ab205851, Clone: EPR4097, 1:50 dilution, Abcam, USA, <https://www.abcam.cn/products/primary-antibodies/alexa-fluor-647-estrogen-receptor-alpha-antibody-epr4097-ab205851.html>)).


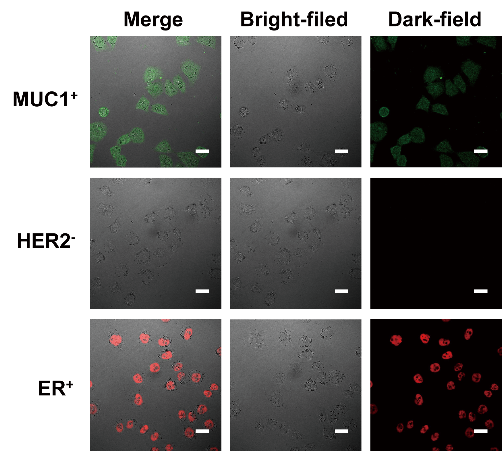


**Supplementary Fig. 15.** Immunofluorescence staining of MUC1/HER2/ER in MCF-7 cells. Scale bar: 20 μm. The representative images are shown from three independent repeats.


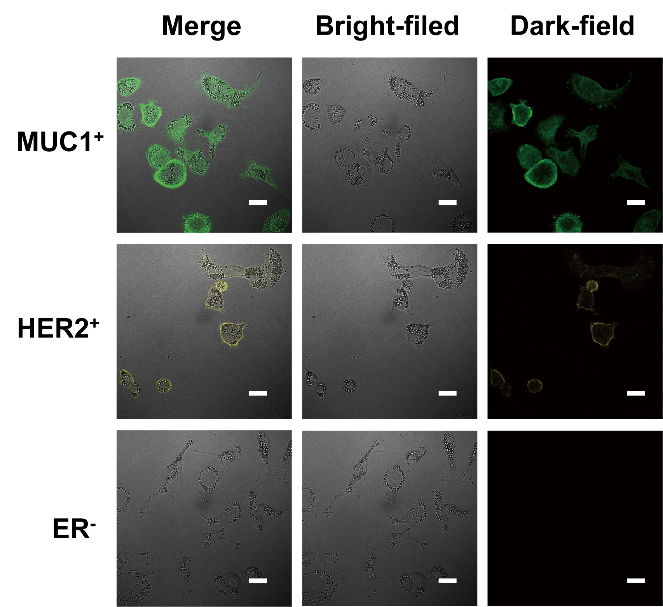


**Supplementary Fig. 16.** Immunofluorescence staining of MUC1/HER2/ER in SK-BR-3 cells. Scale bar: 20 μm. The representative images are shown from three independent repeats.


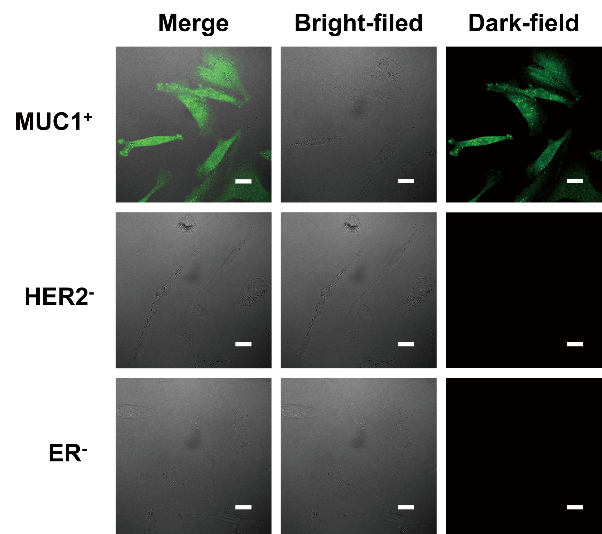


**Supplementary Fig. 17.** Immunofluorescence staining of MUC1/HER2/ER in MDA-MB-231 cells. Scale bar: 20 μm. The representative images are shown from three independent repeats.


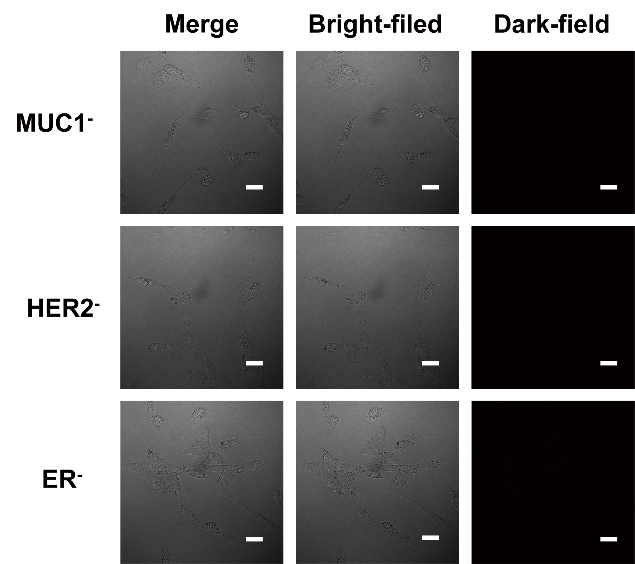


**Supplementary Fig. 18.** Immunofluorescence staining of MUC1/HER2/ER in MCF-10A cells. Scale bar: 20 μm. The representative images are shown from three independent repeats.

**S12. Clinical sample information.**

**Supplementary Table 3. The number of CTCs isolated by this approach and CellSearch® or SE iFISH® from clinical samples.**

| **Sample ID** | **Cancer**  **Stage** | **SE iFISH^®^** | **CellSearch®** | **This work** |
| --- | --- | --- | --- | --- |
|  |  | **Number of CTCs/6 mL** | **Number of CTCs/7.5 mL** | **Number of CTCs/mL** |
| 101 | T2N0M0 IIA | 0 | - | 3 |
| 102 | T2N2M0 IIIA | 0 | - | 10 |
| 103 | TisN0M0 0 | 1 | - | 6 |
| 104 | T2N0M0 IIA | 3 | - | 16 |
| 105 | TisN0M0 0 | 4 | - | 8 |
| 106 | T2N2M0 IIIA | 4 | - | 21 |
| 107 | T2N1M0 IIB | 4 | - | 27 |
| 108 | T1N1M0 IIA | - | 0 | 5 |
| 109 | T1N0M0 IA | - | 4 | 10 |
| 110 | T2N1M1 IV | - | 2 | 18 |


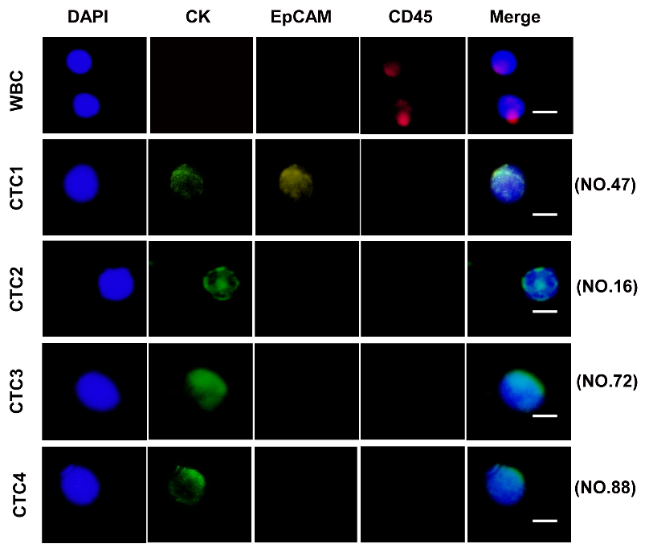


**Supplementary Fig. 19.** Immunofluorescence staining of EpCAM-positive CTC isolated from patient No 47 and EpCAM-negative CTCs isolated from patient No. 16, No. 72 and No. 88, respectively. Scale bar: 10 μm. The representative images are shown from three independent repeats.

**S13. Isolation of CTC mixtures with different EMT subphenotypes by M13-MBs bearing Y-shaped DNA scaffold.**


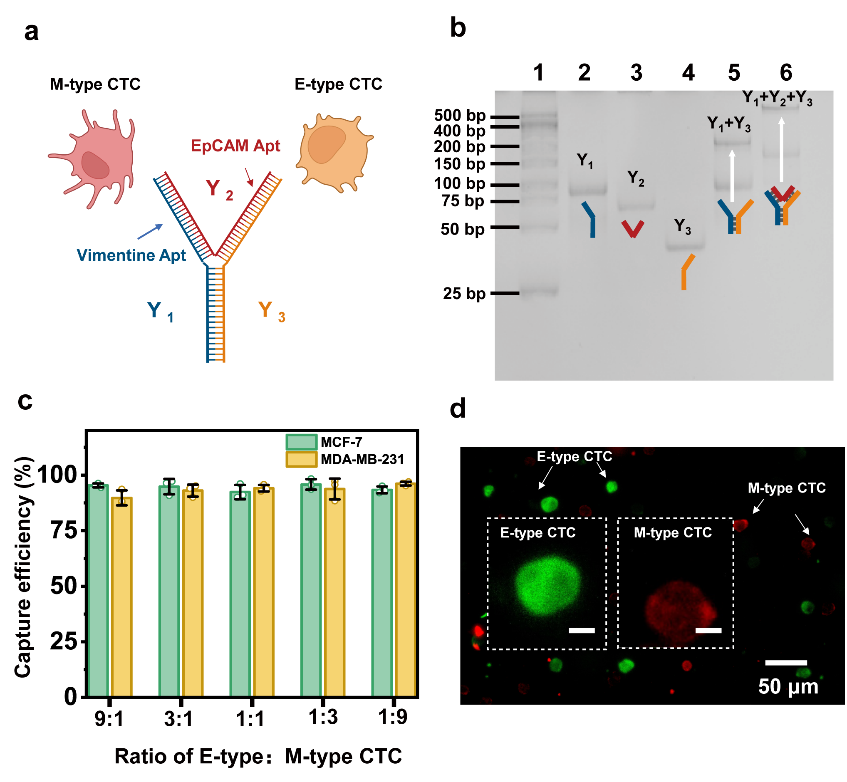


**Supplementary Fig. 20.** The capture and identification of E-type/M-type CTC mixtures by A-f-M13-MB loaded with Y-shaped DNA assemble. (a) Illustration of the construction of Y-shaped DNA assemble. The sequence of anti-vimentine aptamer and anti-EpCAM aptamer were incorporated into scaffold DNA Y_1_ and Y_2_, respectively. The three scaffold DNA Y_1_, Y_2_ and Y_3_ were partially hybridized with each other, thus forming the Y-shaped DNA assemble. Cartoons shown in Supplementary Fig. 20a created with BioRender.com released under a Creative Commons Attribution-NonCommercial-NoDerivs 4.0 International license (https://creativecommons.org/licenses/by-nc-nd/4.0/deed.en). (b) Electrophoresis analysis indicates the successful assembly of the Y-shaped DNA assemble. Lane 1: marker; lane 2: Y_1_; lane 3: Y_2_; lane 4: Y_3_; lane 5: Y_1+3_; lane 6: Y_1+2+3_. (c) The capture efficiency of E-type CTC and M-type CTC by capturing the CTC mixtures by A-f-M13-MB loaded with the Y-shaped DNA assemble (*n* = 3 samples). Error bars represent the mean ± s.d. The ratio of E-type: M-Type=9:1, 3:1, 1:1, 1:3 and 1:9. (d) Representative fluorescence microscope image of the isolated CTC mixture. The ratio of E-type: M-Type=1:1, the E-type CTCs were immunostained by Anti-E Cadherin antibody to emit green fluorescence, whereas the M-type CTCs were labeled with red fluorescence by Anti-N Cadherin antibody. Scale bar: 50 μm. Inserted are enlarged images of E-type CTC and M-type CTC. Scale bar: 10 μm. The representative images in (d) are shown from three independent repeats. The full uncropped scans of the gel images in (b) are provided in the Source Data file. Source data are provided as a Source Data file.

**References**

1. Song, Y. et al. Bioinspired engineering of a multivalent aptamer-functionalized nanointerface to enhance the capture and release of circulating tumor cells. *Angew. Chem. Int. Ed*. **58**, 2236-2240 (2019).

2. Wang, X. Y. et al. M13 phage-based nanoprobe for SERS detection and inactivation of Staphylococcus aureus. *Talanta.* **221**, 121668 (2021).

3. Wang, X., Yang, T., Zhang, X., Chen, M. & Wang, J. In situ-growth of gold nanoparticles on Hg^2+^-binding M13 phages for mercury sensing. *Nanoscale*, **9**, 16728-1673 (2017).
