# Supplementary material for: Harnessing virus flexibility to selectively capture and profile rare circulating target cells for precise cancer subtyping
Source: Nat Commun. 2024 Jul 12;15:5849. doi: 10.1038/s41467-024-50064-y (PMC11239949; doi:10.1038/s41467-024-50064-y)
Supplement: Supplementary file 3 — Description of Additional Supplementary Files [file 41467_2024_50064_MOESM3_ESM.pdf]

Description of Additional Supplementary File

Supplementary Data 1. Information of clinical specimen.

Supplementary Data 2. The raw data of ROC curve.
