# Supplementary material for: Harnessing virus flexibility to selectively capture and profile rare circulating target cells for precise cancer subtyping
Source: Nat Commun. 2024 Jul 12;15:5849. doi: 10.1038/s41467-024-50064-y (PMC11239949; doi:10.1038/s41467-024-50064-y)
Supplement: Supplementary file 6 — Reporting Summary [file 41467_2024_50064_MOESM6_ESM.pdf]

Corresponding author(s): Jian-Yi Li, Hong-Ming Ding, Ting Yang, Chuanbin Mao

Last updated by author(s): May 7, 2024

Reporting Summary

Nature Portfolio wishes to improve the reproducibility of the work that we publish. This form provides structure for consistency and transparency in reporting. For further information on Nature Portfolio policies, see our Editorial Policies and the Editorial Policy Checklist.

Statistics

For all statistical analyses, confirm that the following items are present in the figure legend, table legend, main text, or Methods section.

n/a	Confirmed	
☐	☒	The exact sample size (*n*) for each experimental group/condition, given as a discrete number and unit of measurement
☐	☒	A statement on whether measurements were taken from distinct samples or whether the same sample was measured repeatedly
☐	☒	The statistical test(s) used AND whether they are one- or two-sided<br>*Only common tests should be described solely by name; describe more complex techniques in the Methods section.*
☒	☐	A description of all covariates tested
☒	☐	A description of any assumptions or corrections, such as tests of normality and adjustment for multiple comparisons
☐	☒	A full description of the statistical parameters including central tendency (e.g. means) or other basic estimates (e.g. regression coefficient) AND variation (e.g. standard deviation) or associated estimates of uncertainty (e.g. confidence intervals)
☐	☒	For null hypothesis testing, the test statistic (e.g. *F*, *t*, *r*) with confidence intervals, effect sizes, degrees of freedom and *P* value noted<br>*Give P values as exact values whenever suitable.*
☒	☐	For Bayesian analysis, information on the choice of priors and Markov chain Monte Carlo settings
☒	☐	For hierarchical and complex designs, identification of the appropriate level for tests and full reporting of outcomes
☒	☐	Estimates of effect sizes (e.g. Cohen's *d*, Pearson's *r*), indicating how they were calculated

Our web collection on statistics for biologists contains articles on many of the points above.

Software and code

Policy information about availability of computer code

Data collection	Dissipative particle dynamics simulations were carried out using the modified software package Lammps (12 Dec 2018). The force induced deformation data of M13 nanofibers with different stiffnesses simulations were collected by Numerical simulations using COMSOL (version 5.6, COMSOL Ltd, USA). The mean distance and migration speed of cells before and after isolation and re-culture were collected using Image J software(version 1.53t) with Manual Tracking plugin.
Data analysis	IBM SPSS Statistics software (version 19.0) was used to test the difference for diagnosis stages from different CTCs using a two-tailed t-test. Confusion matrix for subtype diagnosis by CTC molecular profiling method was drawn by Origin software (version 2021). Receiver operating characteristic (ROC) curve was plotted using the MedCalc statistical software(version 20. 010), presenting the diagnostic accuracy. The movement trajectory of cells before and after isolation and re-culture was analyzed using Matlab software(version R2020b).The results of dissipative particle dynamics simulations were analyzed by Graph pad prism 9 (version 9.3.0).

For manuscripts utilizing custom algorithms or software that are central to the research but not yet described in published literature, software must be made available to editors and reviewers. We strongly encourage code deposition in a community repository (e.g. GitHub). See the Nature Portfolio guidelines for submitting code & software for further information.

Data

Policy information about availability of data

All manuscripts must include a data availability statement. This statement should provide the following information, where applicable:

- Accession codes, unique identifiers, or web links for publicly available datasets
- A description of any restrictions on data availability
- For clinical datasets or third party data, please ensure that the statement adheres to our policy

The data that support the findings of this study are available within the paper and Supplementary Information. Source data are provided with this paper.

Research involving human participants, their data, or biological material

Policy information about studies with human participants or human data. See also policy information about sex, gender (identity/presentation), and sexual orientation and race, ethnicity and racism.

Reporting on sex and gender	In this study, clinical samples were collected based on biological sex. Sex of participants was defined based on self-report. As breast cancer is mostly suffered by female patients, most of the participants involved in this study were female (95%). Considering the possibility of male patients that suffer from benign breast disease, the cohort involved four healthy male donors and one male benign patient. The sex information has been indicated clearly in the Excel file "Supplementary Table 3. Information of clinical specimen".
Reporting on race, ethnicity, or other socially relevant groupings	Neither race nor ethnicity were included in our study. All participants in this study were Asian.
Population characteristics	For the diagnostics of breast cancer (BC), 100 participants were enrolled, with ages ranging from 20 to 82 years, including 90 patients that had been diagnosed as BC (n=56) or benign breast disease (n=34), and 10 healthy donors. All patients received no neoadjuvant treatment or surgery. Relevant information on the human participants is presented in the Excel file "Supplementary Table 3. Information of clinical specimen".
Recruitment	All participants were recruited from Liaoning Cancer Hospital. Only patients with definite information of sex, age, and pathological diagnosis were recruited. The study complied with all relevant ethical regulations and was approved by the Ethics Committee of both Northeastern University, China (No. NEU-EC-2021B020S) and Liaoning Cancer Hospital (20211035). All individuals were anonymous, and only gender, age, pathological diagnosis, treatment plan and treatment response were recorded. No self-selection criteria bias for patient populations was anticipated. The consent to publish the participants' information regarding their age and sex were obtained by the co-author (J.Y.L).
Ethics oversight	The clinical specimens were evaluated in compliance with the relevant regulations and institutional guidelines, with approval by the ethical committee of both Northeastern University, China and Liaoning Cancer Hospital.

Note that full information on the approval of the study protocol must also be provided in the manuscript.

Field-specific reporting

Please select the one below that is the best fit for your research. If you are not sure, read the appropriate sections before making your selection.

☒ Life sciences ☐ Behavioural & social sciences ☐ Ecological, evolutionary & environmental sciences

For a reference copy of the document with all sections, see nature.com/documents/nr-reporting-summary-flat.pdf

Life sciences study design

All studies must disclose on these points even when the disclosure is negative.

Sample size	For the diagnostics of breast cancer (BC), 100 participants were enrolled, with ages ranging from 20 to 82 years, including 90 patients that had been diagnosed as BC (n=56) or benign breast disease (n=34), and 10 healthy donors. For the subtyping of BC, all 56 BC patients were involved.
Data exclusions	No data were excluded.
Replication	The experiments were performed in three independent replicates as properly indicated in the figure displays or figure legends. The replications had similar and consistent results in our research.
Randomization	Samples were randomly allocated.
Blinding	The experiments were blinded to the operator.

Reporting for specific materials, systems and methods

We require information from authors about some types of materials, experimental systems and methods used in many studies. Here, indicate whether each material, system or method listed is relevant to your study. If you are not sure if a list item applies to your research, read the appropriate section before selecting a response.

Materials & experimental systems

n/a	Involved in the study
☐	☒ Antibodies
☐	☒ Eukaryotic cell lines
☒	☐ Palaeontology and archaeology
☒	☐ Animals and other organisms
☒	☐ Clinical data
☒	☐ Dual use research of concern
☒	☐ Plants

Methods

n/a	Involved in the study
☒	☐ ChIP-seq
☒	☐ Flow cytometry
☒	☐ MRI-based neuroimaging

Antibodies

Antibodies used

Alexa Fluor® 647 Anti-Estrogen Receptor alpha antibody from Abcam (Cat. #: ab205851, Clone: EPR4097, (1:50 dilution), https://www.abcam.cn/products/primary-antibodies/alexa-fluor-647-estrogen-receptor-alpha-antibody-epr4097-ab205851.html),
Alexa Fluor® 488 Anti-HER2 antibody from Abcam (Cat. #: ab237060, clone: EP2324Y, (1:100 dilution), https://www.abcam.cn/products/primary-antibodies/alexa-fluor-488-erbb2--her2-phospho-y877-antibody-ep2324y-ab237060.html),
Alexa Fluor® 488 Anti-MUC1 antibody from Abcam (Cat. #: ab196443, clone: EPR1023, (1:50 dilution), https://www.abcam.cn/products/primary-antibodies/alexa-fluor-488-muc1-antibody-epr1023-ab196443.html),
Anti-N Cadherin antibody from Abcam (Cat. #: ab98952), clone: 5D5, (1:200 dilution), https://www.abcam.cn/products/primary-antibodies/n-cadherin-antibody-5d5-intercellular-junction-marker-ab98952.html),
Anti-E Cadherin antibody from Abcam (Cat. #: ab40772, clone: EP700Y, (1:1000 dilution), https://www.abcam.cn/products/primary-antibodies/e-cadherin-antibody-ep700y-intercellular-junction-marker-ab40772.html),
Anti-Estrogen Receptor alpha Antibody from Abcam (Cat. #: ab32063, clone: E115, (1/500 dilution), https://www.abcam.cn/products/primary-antibodies/estrogen-receptor-alpha-antibody-e115-chip-grade-ab32063.html),
Anti-GAPDH antibody- Loading Control from Abcam (Cat. #: ab9485, clone: pAb, (1:2500 dilution), https://www.abcam.cn/products/primary-antibodies/gapdh-antibody-loading-control-ab9485.html),
HRP Conjugated AffiniPure Goat Anti-rabbit IgG (H+L) from Boster (Cat. #: BA10541, clone: NA, (1: 8000 dilution), http://boster.com/index/products/productsDetail?goods_sn=BA1054),
Goat Anti-Rabbit IgG H&L (Alexa Fluor® 488) from Abcam (Cat. #: ab150077, clone: NA, (1:200 dilution), https://www.abcam.cn/products/secondary-antibodies/goat-rabbit-igg-hl-alexa-fluor-488-ab150077.html)
Goat Anti-Mouse IgG H&L (Alexa Fluor® 594) (Cat. #: ab150116, clone: NA, (1:200 dilution), https://www.abcam.cn/products/secondary-antibodies/goat-mouse-igg-hl-alexa-fluor-594-ab150116.html).

Validation

All antibodies in this study were provided by commercial suppliers and validated by the references mentioned on the supplier's official website for immunofluorescence staining and Western blotting applications.

Eukaryotic cell lines

Policy information about cell lines and Sex and Gender in Research

Cell line source(s)

MCF-7 (Cat. #: TCHu 74), HepG2 (Cat. #: TCHu 72) and A549 (Cat. #: TCHu150) cell lines were obtained from Stem Cell Bank, Chinese Academy of Sciences. MCF-10A (Cat. #: CL-0212), MDA-MB-231 (Cat. #: CL-0150B), Ramos (Cat. #: CL-0483) and SK-Hep-1 (Cat. #: CL-0525) cell line cell lines were purchased from Procell (Wuhan, China).

Authentication

The cell lines were directly used as received and none of the cell lines used were authenticated by ourselves.

Mycoplasma contamination

We run mycoplasma test to ensure the cells were free from mycoplasma contamination.

Commonly misidentified lines (See ICLAC register)

No misidentified cell lines were used in this study.

Plants

Seed stocks	*Report on the source of all seed stocks or other plant material used. If applicable, state the seed stock centre and catalogue number. If plant specimens were collected from the field, describe the collection location, date and sampling procedures.*
Novel plant genotypes	*Describe the methods by which all novel plant genotypes were produced. This includes those generated by transgenic approaches, gene editing, chemical/radiation-based mutagenesis and hybridization. For transgenic lines, describe the transformation method, the number of independent lines analyzed and the generation upon which experiments were performed. For gene-edited lines, describe the editor used, the endogenous sequence targeted for editing, the targeting guide RNA sequence (if applicable) and how the editor was applied.*
Authentication	*Describe any authentication procedures for each seed stock used or novel genotype generated. Describe any experiments used to assess the effect of a mutation and, where applicable, how potential secondary effects (e.g. second site T-DNA insertions, mosiacism, off-target gene editing) were examined.*
